# Stereocontrolled synthesis and nuclear magnetic resonance analysis of boranophosphate/phosphate chimeric oligonucleotides

**DOI:** 10.1098/rsos.241797

**Published:** 2025-04-09

**Authors:** Kiyoshi Kakuta, Taiichi Sakamoto, Hajime Sato, Kazuki Sato, Takeshi Wada

**Affiliations:** ^1^ Department of Medicinal and Life Sciences, Tokyo University of Science, Noda, Chiba, Japan; ^2^ Department of Life Science, Chiba Institute of Technology, Narashino, Chiba, Japan; ^3^ BioSpin Division, Bruker Japan K.K., Yokohama, Kanagawa, Japan

**Keywords:** stereoselective synthesis, boranophosphate DNA, nuclear magnetic resonance analysis

## Abstract

This report describes the stereoselective synthesis of boranophosphate/phosphate (PB/PO) chimeric oligodeoxynucleotides (ODNs) and the nuclear magnetic resonance (NMR) analysis of oligomers containing PB linkages. This stereoselective synthesis involved dimer building blocks containing highly stereopure boranophosphotriester linkages, which enabled the synthesis of PB/PO chimeric ODNs via the standard phosphoramidite method. The stereochemistry of dinucleoside and trinucleoside PBs was confirmed by nuclear Overhauser effect spectroscopy (NOESY) experiments. In the NOESY experiments, the stereochemistry was determined from the correlation of the protons of the borane (BH_3_) group with those of neighbouring 2'-deoxyribose moieties or nucleobases. Thus, the stereochemistry of the PB linkages within the oligomer was determined, and the stereopurity was also confirmed. A PB linkage was introduced into ODNs with retention of the *P*-configuration of the dimer building blocks without any loss of stereopurity during solid-phase synthesis. This synthetic approach is expected to be a reliable method for introducing PB linkages with precise stereochemistry. Additionally, detailed analysis of the PB linkages via NMR allowed accurate determination of the stereochemistry.

## 1. Introduction

Antisense therapy employs therapeutic remedies that target messenger RNA (mRNA) with antisense oligonucleotides (ASOs) [1]. The number of approved ASO drugs has increased drastically since 2016 [2]. ASOs can regulate mRNA expression through a variety of mechanisms, including ribonuclease H (RNase H)-mediated mRNA degradation and exon skipping and inclusion by binding to target pre-mRNAs and controlling splicing [1].

Phosphorothioate (PS) modification of the phosphorus atoms in ASOs is common [1]. PS-ASOs exhibit substantial nuclease resistance, favourable pharmacokinetic activity and sufficient efficacy. However, some PS-ASOs induce cytotoxicity as a result of interactions with certain kinds of proteins. Thus, the development of potent ASOs with good safety and efficacy remains challenging, hindering the development of therapeutic ASOs [3–5]. The development of alternative *P*-modifications is a promising strategy to enhance the therapeutic potential of ASOs [6–8]. Boranophosphate (PB) modification, in which a non-bridging oxygen within a phosphodiester bond is replaced with a borane (BH_3_) group, is a candidate for the preparation of ASOs. PB-oligodeoxynucleotides (PB-ODNs) induce the RNase H-mediated cleavage of complementary RNA and have higher nuclease resistance than PS-ODNs [9]. Notably, PB-ODNs exhibit low cytotoxicity [10].

**Figure 1.**
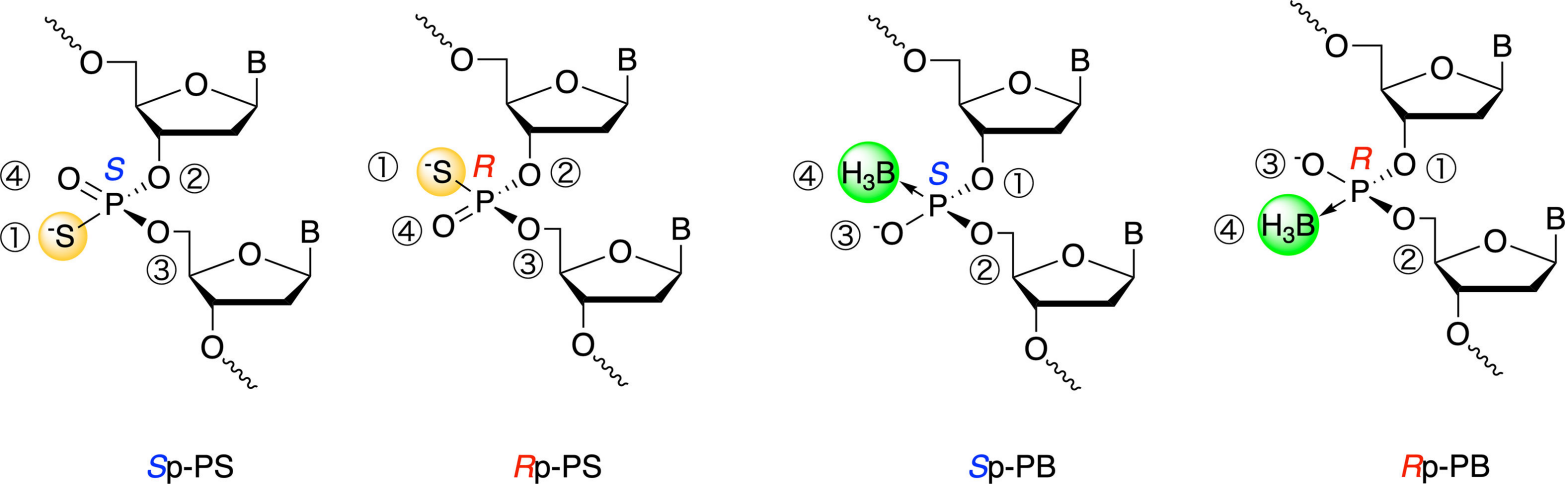
Structures and stereochemistry of *P*-modified ODNs.

Furthermore, *P*-modifications, including the introduction of PS and PB linkages, result in the formation of two diastereomers per modification. Notably, PS and PB diastereomers with the same orientation have opposite Prelog designations because of the atomic priority order: S > O > B (figure 1). These diastereomers exhibit different physico-chemical and biological properties, such as the ability to form duplexes with complementary RNA, nuclease resistance and the ability to induce RNase H-mediated cleavage [11–13]. However, the effects of the stereocontrol of PB linkages on the physico-chemical and biological properties of PB-ODNs are largely unknown. To elucidate the relationship between the stereochemistry and properties of PB-ODNs, the development of a synthetic method for precise stereo-chemical control and an analytical method to investigate stereochemistry and stereopurity is crucial.

In this study, we aimed to synthesize 12-mer ODNs containing a single stereopure PB linkage (PB/phosphate (PO) chimeric ODNs). First, a synthetic method for the stereoselective introduction of a PB linkage was developed. Subsequently, the stereochemistry and purity of the synthesized ODNs were comprehensively analysed by nuclear magnetic resonance (NMR) measurements.

**Scheme 1.**
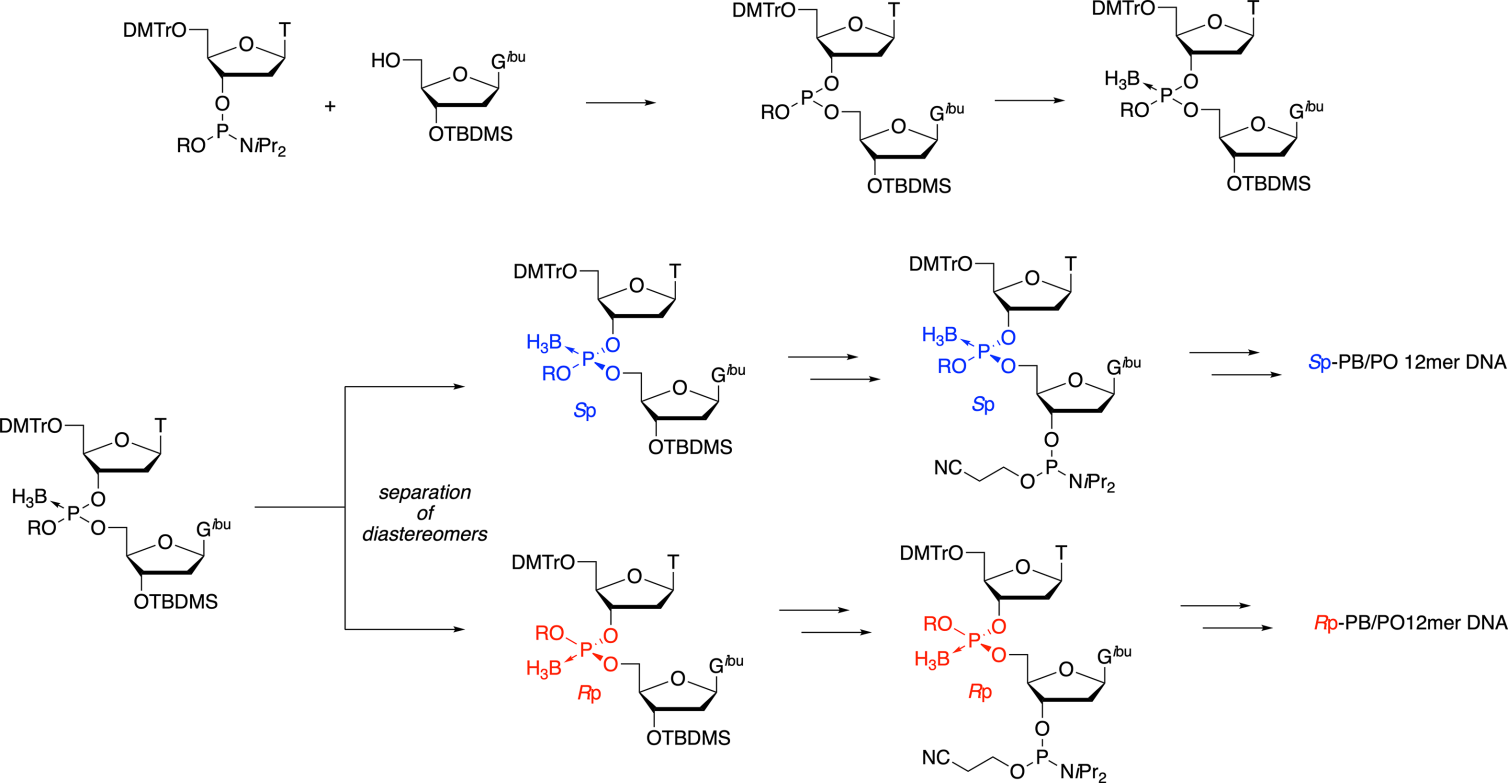
Outline of the synthetic strategy for the synthesis of stereoregulated PB/PO chimeric ODNs.

Generally, ODNs are synthesized by solid-phase synthesis using the phosphoramidite method [14,15]. The amino groups of nucleobases are typically protected by acyl groups to prevent undesired nucleobase phosphitylation. Theoretically, PB-ODNs can be synthesized by boronation of a phosphite triester intermediate formed via a condensation reaction. However, a portion of the amide groups on the nucleobases are irreversibly reduced to alkylamino groups via a serious side reaction during the boronation reaction [16]. Therefore, the synthesis of PB-ODNs via phosphoramidite chemistry is challenging. In this study, we employed dimer building blocks containing a stereopure internucleotidic boranophosphsphotriester linkage for the solid-phase synthesis of PB/PO chimeric ODNs. After the synthesis of a dinucleoside boranophosphotriester, which existed as a mixture of diastereomers, these diastereomers were separated to obtain stereodefined products (scheme 1). Then, a phosphoramidite moiety was introduced to afford dimer building blocks. Because the BH_3_ group was previously introduced on an internucleotidic linkage, the utilization of these dimer building blocks afforded PB/PO chimeric ODNs containing stereocontrolled PB linkages without a boronation step, thereby enabling the use of the phosphoramidite method. Although PB/PO chimeric ODNs were synthesized without boronation via solid-phase synthesis, the most challenging step of this synthetic strategy was boronation without reduction of the amide groups to synthesize the dimer building blocks.

Additionally, NMR analysis was employed to determine the stereochemistry of the synthesized ODNs and quantify their stereopurity. The stereochemistry of *P*-modified ODNs, such as methylphosphonate and alkyl phosphotriester, was determined by nuclear Overhauser effect spectroscopy (NOESY) or rotating-frame nuclear Overhauser effect spectroscopy (ROESY). Furthermore, nuclear Overhauser effect (NOE) correlation of the signals attributed to the methyl group on the phosphorus atom, and the protons of the 2'-deoxyribose moiety allowed determination of the stereochemistry of the phosphorus atom in methylphosphonate [17–19].

Johnson *et al.* analysed the structure of DNA/RNA duplexes containing a single *S*p or *R*p PB linkage in the DNA strand via NMR measurements [20]. They determined the stereochemistry of the PB linkage by means of an enzyme digestion assay in which the PB linkage was cleaved stereoselectively. The spatial information obtained from the NOESY experiments was in a good agreement with the results of the enzyme digestion assay.

However, during NMR analysis of PB derivatives, it is difficult to observe the NOE of the protons in the BH_3_ groups because of the properties of the boron atoms, which cause broadening of the ^1^H NMR and ^31^P NMR signals [21]. There are two stable isotopes of boron (^10^B : ^11^B = 19.58 : 80.42), and each boron atom exhibits complex coupling with the other atoms in NMR spectra. The ^10^BH_3_ and ^11^BH_3_ signals in ^1^H NMR spectra split into seven and four signals, respectively, because the spin quantum numbers of ^10^B and ^11^B are 3 and 3/2, respectively. Similarly, the PB signal in ^31^P NMR spectra is broad because of coupling between ^31^P atom and ^10^B or ^11^B atom. These properties make the NMR signals of PB derivatives complicated.

In this study, we investigated the stereochemistry and purity of dimers, trimers, single-stranded DNAs and double-stranded DNA/RNA duplexes through comprehensive NMR analysis using double- and triple-resonance NMR spectroscopy.

## 2. Results and discussion

### 2.1. Chemical synthesis

#### 2.1.1. Solid-phase synthesis of dimers and trimers

**Figure 2.**
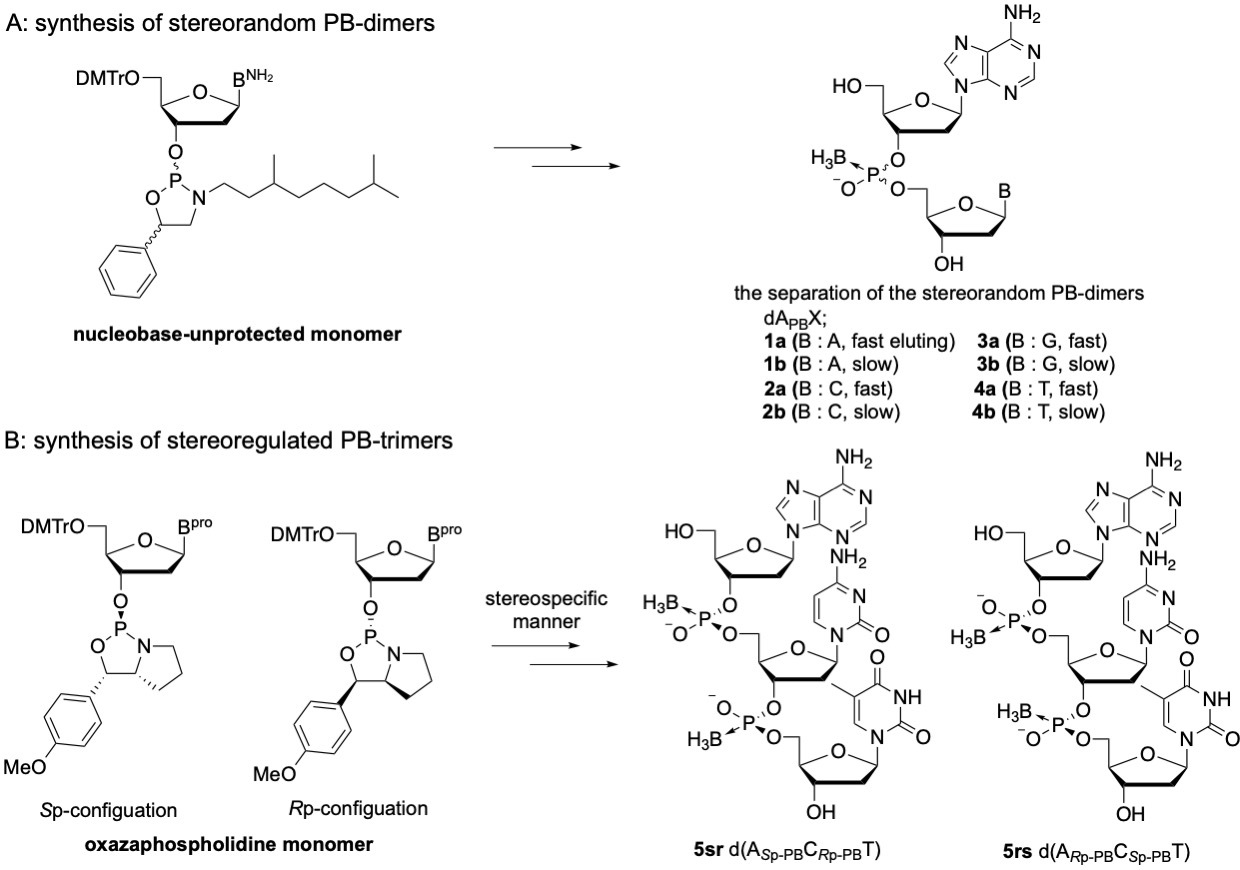
Solid-phase synthesis of dimers **1-4ab** and trimers **5sr** and **5rs**.

First, four kinds of dinucleoside PBs, 5ʹ-dA_PB_X−3ʹ (X = A, C, G, T), were synthesized as simple model compounds to analyse the structures of PB-ODNs. The synthesis was carried out by the use of diastereomixtures of nucleobase-unprotected oxazaphospholidine derivatives developed in our laboratory [22], which afforded diastereomixtures of a dinucleoside PB (figure 2A). Two reverse-phase (RP) high-performance liquid chromatography (HPLC) peaks were attributed to the diastereomers in the crude mixtures after typical ammonia treatment. The fractions corresponding to each peak were collected to isolate each diastereomer. We referred to the fast-eluting dimer and the slow-eluting dimer as the ‘a’ and ‘b’ isomers of dA_PB_X, respectively, on the basis of the HPLC retention times.

Next, we synthesized two diastereomers of trinucleoside PBs: 5ʹ-d(A*_S_*_p-PB_C*_R_*_p-PB_T)−3ʹ and 5ʹ-d(A*_R_*_p-PB_C*_S_*_p-PB_T)−3ʹ. Because trinucleoside PBs have four diastereomers that are difficult to separate, we synthesized the trimers in a stereoselective manner using the oxazaphospholidine method (figure 2B) [12]. Oxazaphospholidine is a cyclic phosphoramidite derivative with a chiral auxiliary group, and we have previously reported the stereoselective synthesis of PB-DNAs via this method. It was confirmed via an enzyme digestion assay that an *R*p- or *S*p-PB-DNA was generated by using an *R*p- or *S*p-oxazaphospholidine monomer, respectively [13]. All the dimers and trimers were characterized by electrospray ionization mass spectrometry (ESI-MS) and NMR spectroscopy. The results of the NMR analysis are discussed hereafter.

#### 2.1.2. Synthesis of dimer building blocks with a stereopure boranophosphotriester linkage

Next, for the solid-phase synthesis of stereoregulated PB/PO chimeric ODNs, namely, 5ʹ-d(GCATT_PB_GGTATTC)−3ʹ, whose sequences were antisense relative to the apoB protein mRNA [23], we first synthesized dimer building blocks (5ʹ-T_PB_G−3ʹ) with a stereopure boranophosphotriester linkage. To synthesize the boranophosphotriester derivative, a methyl group was used as a protecting group of the boranophosphotrister moiety [15].

**Scheme 2.**
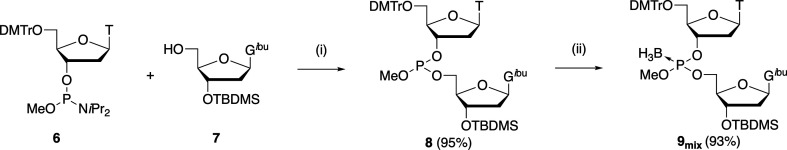
Synthesis of boranophosphotriester **9mix**. (i) **7** (1.1 equiv), DCI (2.0 equiv), MeCN, room temperature (rt), 10 minutes, 95%; (ii) 2-picoline-BH_3_ (4 equiv), THF, rt to 40°C, 1 day, 93%.

According to Scheme 2, 5ʹ-*O*-dimethoxytrityl (DMTr)−3ʹ-phosphoramidite thymidine derivative **6** containing a methyl protecting group on the phosphoramidite moiety was condensed with the 5ʹ-hydroxy group of *N*^2^-isobutyryldeoxyguanosine derivative **7** in the presence of 4,5-dicyanoimidazole (DCI) as an acidic activator, followed by boronation of phosphite intermediate **8**, which afforded dinucleoside boranophosphotriester derivative **9mix**. The most challenging step of this strategy was the final step: as described previously, the acyl protecting group on the amino group of a nucleobase was prone to reduction by a boronation reagent. The rate of the side reaction largely depended on the boronation reagent, the equivalents present and the nucleobase [16,24]. Thus, we investigated which boronation conditions could afford the boranophosphotriester without side reactions involving the nucleobase. Four boronation reagents, namely, pyridine-BH_3_, 2-picoline-BH_3_, 2,6-lutidine-BH_3_ and BH_3_-THF complexes, were examined via small-scale synthesis of **9mix** (table 1). When the pyridine-BH_3_ complex was used, boronation proceeded with a yield of only 80% after 20 hours at 40°C. In contrast, the use of the 2-picoline-BH_3_ complex resulted in complete reaction after 20 hours. Furthermore, the 2,6-lutidine-BH_3_ and BH_3_-THF complexes reacted immediately, demonstrating the fastest rates among the tested reagents. However, the BH_3_-THF complex generated a by-product in approximately 60% yield, which exhibited a lower retention factor (Rf) value on thin-layer chromatography (TLC) than the target product, probably because of reduction of the amide group on the nucleobase. Conversely, the formation of such by-products was not observed with the other BH_3_ complexes.

**Table 1.** Synthesis of dinucleoside boranophosphotriesters.

entry	BH_3_ complex	conversion to boranophosphotriester (%)^a^			by-product^b^
		30 min	8 h	20 h	
1	pyridine-BH_3_	2	n.d.	80	n.d.
2	2-picoline-BH_3_	7	96	100	n.d.
3	2,6-lutidine-BH_3_	100	—	—	n.d.
4	BH_3_-THF	100	—	—	detected^c^

^a^ Determined by ^31^P NMR (electronic supplementary material, figure S1): integral ratios of (compound **9**/(compound **8** + compound **9**)).

^b^ Determined by TLC.

^c^ A by-product with a lower Rf value determined by TLC than the target product was detected.

A study on the reactivity of the BH_3_ complexes demonstrated that the affinity of ligands to BH_3_ significantly influenced the efficiency of boronation [24]. BH_3_-THF exhibited the fastest exchange rate from the ligand to the phosphite triester because the relative affinity of THF to BH_3_ was the lowest. Although the use of 2-picoline-BH_3_ resulted in a slow reaction, we selected this complex as an optimum boronation reagent because it prevents the occurrence of side reactions, ensures complete boronation on the phosphite triester and is commercially available, while 2,6-lutidine-BH_3_ is not. Notably, 2-picoline, the ligand generated after boronation, did not cause deboronation of the boranophosphotriester derivative when the solvents were removed under reduced pressure. Deboronation has been reported to occur easily in the presence of pyridine [25].

**Scheme 3.**
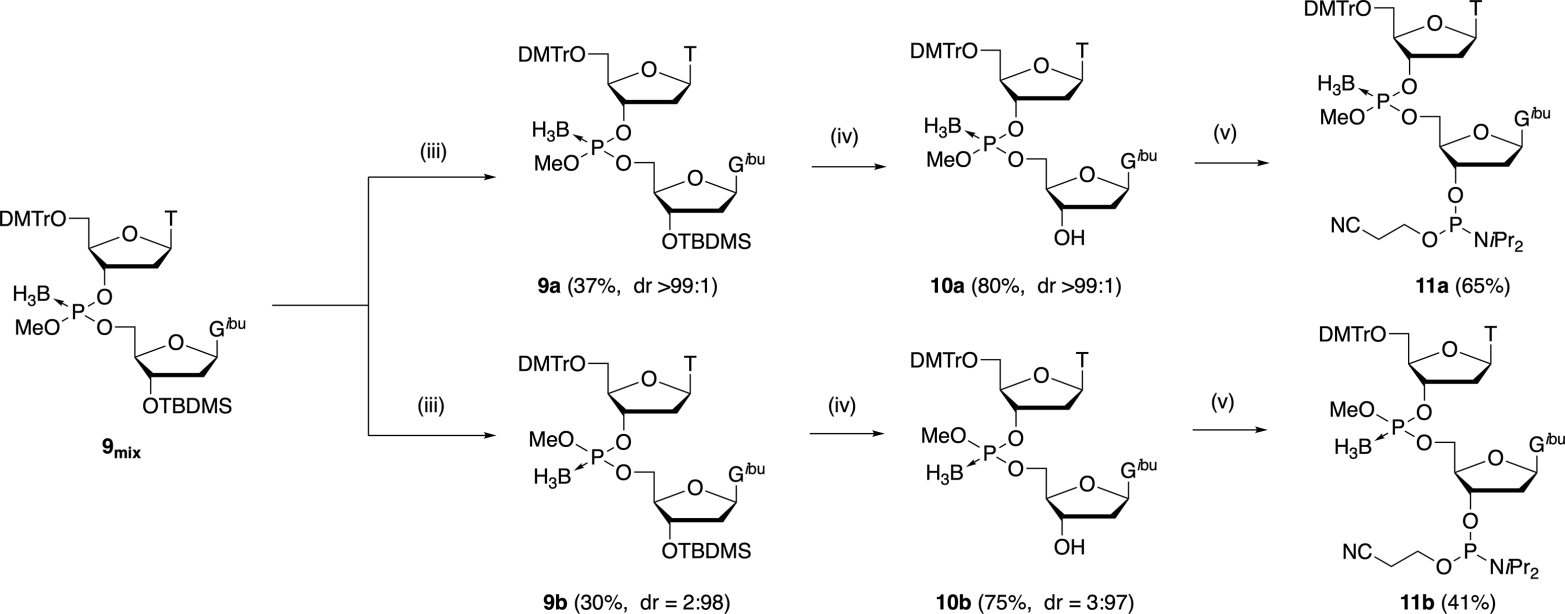
Separation of diastereomers and synthesis of dimer building blocks **11a** and **11b**. (iii) Silica gel column chromatography once, 37%, *dr* > 99 : 1 for **9a**, silica gel column chromatography twice, 30%, *dr* = 2 : 98 for **9b**; (iv) TEA•3HF (8 equiv), THF, rt, 1 day, 80%, *dr* > 99 : 1 for **10a**, 75%, *dr* = 3 : 97 for **10b**; (v) 2-cyanoethyl diisopropyl chlorophosphoramidite (2.5 equiv), TEA (5.0 equiv), CH_2_Cl_2_, rt, 30 minutes, 65% for **11a**, 41% for **11b**.

When the optimized reaction conditions were used, the protected dinucleoside boranophosphotriester **9mix** was obtained in 93% isolated yield. Then, the diastereomers were separated via silica gel column chromatography (Scheme 3). As shown in electronic supplementary material, figure S2, there were two peaks in the chromatography profile, and the fractions corresponding to each peak were collected to isolate each diastereomer with high stereopurity, as determined from the integral ratios of the H3' signals of T (fast-eluting isomer: diastereomeric ratio (*dr*) > 99 : 1, slow-eluting isomer: *dr* = 2 : 98; electronic supplementary material, figure S3). We referred to the fast-eluting isomer as **9a** or the slow-eluting isomer as **9b** for clarification. Compounds **9a** or **9b** were isolated in 37 or 30% yields after performing column chromatography once or twice, respectively (Scheme 3).

Next, the *tert*-butyldimethylsilyl group was removed by using trimethylamine trihydrofluoride (TEA•3HF). Compounds **10a** and **10b** were obtained with high stereopurities (**10a**: *dr* > 99 : 1, **10b**: *dr* = 3 : 97). Subsequent phosphitylation of the 3'-hydroxy groups afforded dimer building blocks bearing a stereopure boranophosphotriester linkage, **11a** and **11b**, in modest yields (Scheme 3).

#### 2.1.3. Solid-phase synthesis of T_PB_G using dimer building blocks with a stereopure boranophosphotriester linkage

**Scheme 4.**
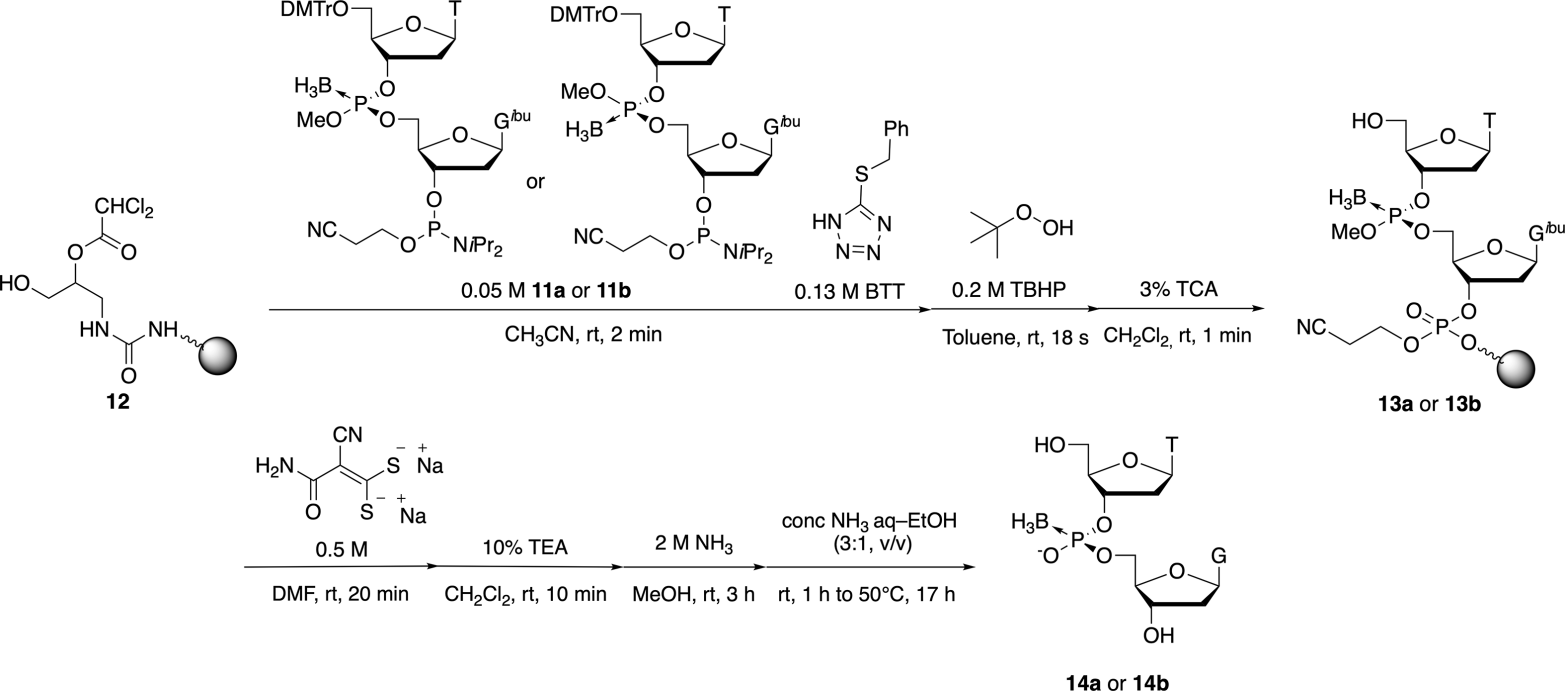
Solid-phase synthesis of T_PB_G using dimer building blocks with a stereopure boranophosphotriester linkage.

Next, to confirm that the stereochemistry and purity of dimer building blocks **11a** and **11b** were retained during the solid-phase synthesis, we conducted the solid-phase synthesis of T_PB_G (**14a** and **14b**) from **11a** and **11b**, with Universal Support III (USIII, Glen Research, VA) [26] as a solid support (Scheme 4).

Dimer building blocks **11a** and **11b** were condensed with a hydroxy group on the universal support **12**, and 5-benzylthiotetrazole (BTT) was used as an acidic activator. The resultant phosphite triester was oxidized by treatment with 0.2 M *tert*-butyl hydroperoxide (TBHP) in toluene. Then, the DMTr group on the 5'-hydroxy group was removed under acidic conditions with 3% trichloroacetic acid (TCA). After deprotection of the methyl group by treatment with disodium 2-carbamoyl−2-cyanoethylene−1,1-dithiolate [27] and the cyanoethyl groups by treatment with Et_3_N, 2 M NH_3_ in a MeOH solution was used to cleave the universal linker. Finally, the nucleobase-protecting groups were removed with a mixture of concentrated NH_3_ (aq)–EtOH (3 : 1, v/v), and the mixture was analysed by RP-HPLC.

The single peaks shown in electronic supplementary material, figure S6 were attributed to target compound T_PB_G on the basis of the ESI-MS results. Compound **14a** and **14b** isomers were obtained from dimer building blocks **11a** and **11b**, respectively. The stereopurities of **14a** and **14b** were 99 and 97%, respectively, as confirmed by the area ratio defined by product/(product + compound with opposite stereochemistry). The successful synthesis of T_PB_G **14a** and **14b** indicated that the boranophosphotriester linkage was stable under oxidation conditions in the presence of 0.2 M TBHP. Herein, we demonstrated that the dimer building blocks were applicable to the synthesis of stereopure PB derivatives.

#### 2.1.4. Solid-phase synthesis of oligodeoxynucleotides containing a stereopure boranophosphate linkage

**Scheme 5.**
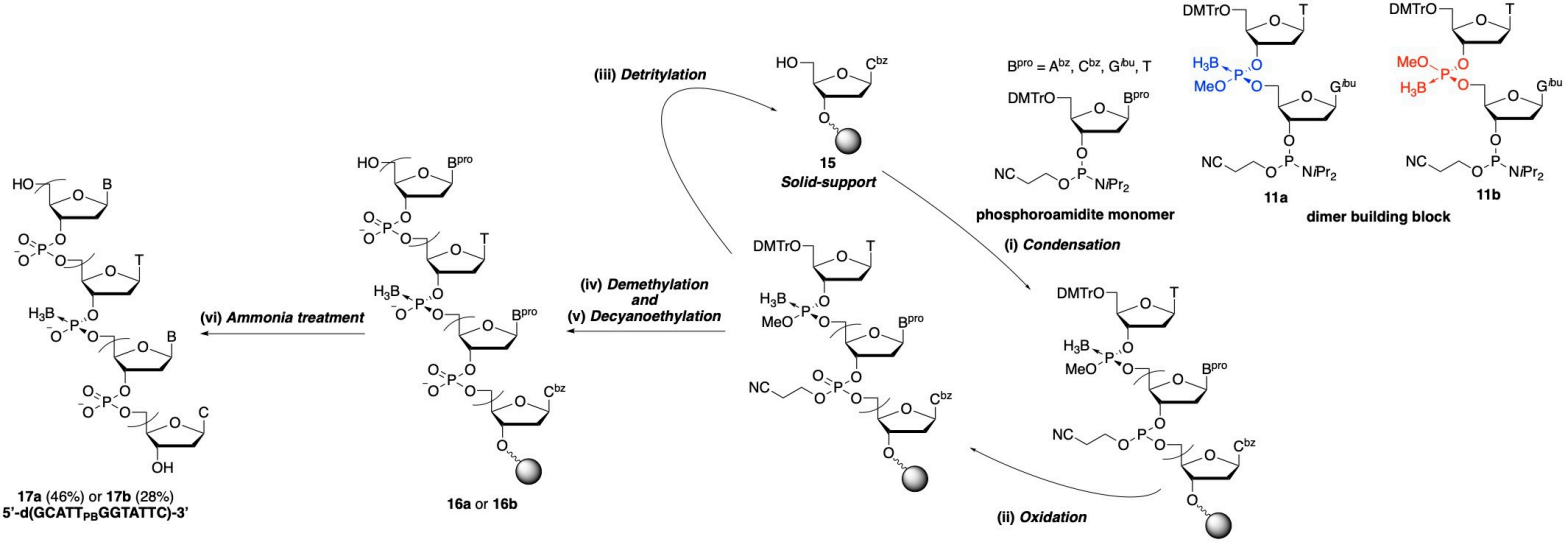
Synthesis of dodecamers containing a stereopure PB linkage. (i) 0.05 M **11a** or **11b** with A^bz^, C^bz^, G*^i^*^bu^ or T phosphoramidite monomers, 0.13 M BTT, MeCN, 1 minute × 2; (ii) 0.2 M TBHP, toluene, 18 seconds; (iii) 3% TCA, CH_2_Cl_2_, 1 minute; (iv) 0.5 M disodium 2-carbamoyl−2-cyanoethylene−1,1-dithiolate, dimethylformamide (DMF), 20 minutes; (v) 10% Et_3_N, CH_2_Cl_2_ 10 minutes; (vi) conc NH_3_ (aq)–EtOH (3 : 1, v/v), rt (1 h) to 50°C (17 h).

Next, ODNs d(GCATT_PB_GGTATTC) containing a stereopure PB linkage **17a** and **17b** were synthesized using dimer building blocks **11a** and **11b** (Scheme 5). Dimer building blocks **11a** or **11b** and the A, C, G and T phosphoramidite monomers were used for condensation reactions. A controlled pore glass support loaded with *N*^4^-benzoyldeoxycytidine via succinyl linker **15** was used as a solid support, and the oligomers were synthesized by repeated cycles of condensation, oxidation and detritylation to achieve chain elongation. After removal of the methyl and cyanoethyl groups, removal of nucleobase-protecting groups and cleavage of the succinyl linker were conducted in a mixture of a concentrated NH_3_ (aq)–EtOH (3 : 1, v/v). The reaction mixtures were analysed by ion pair RP ultraperformance liquid chromatography (IP-RP ultra-performance liquid chromatography (UPLC)). As seen from the UPLC profiles (electronic supplementary material, figure S7), the area ratio of the 10-mer without the T_PB_G and **17** (12-mer) was 81 : 19–82 : 18, indicating that the condensation reaction using dimer building block **14** did not occur efficiently. The condensation efficiencies observed in this study were comparable to those of other types of phosphoramidite dimer building blocks [28].

After purification by IP-RP UPLC, sufficient quantities (109 and 57 nmol, respectively) of each ODN (**17a** and **17b**) were obtained for NMR measurements. The yields of each isomer (**17a** from **11a**, **17b** from **11b**) were 46 and 28%, respectively. The yields of the oligomers synthesized by this method were the highest among those achieved with all synthetic methods used to synthesize ODNs with PB linkages containing four nucleobases in our laboratory [11,12,22,29–31].

### 2.2. Nuclear magnetic resonance measurements

#### 2.2.1. Analysis of dimers

**Figure 3.**
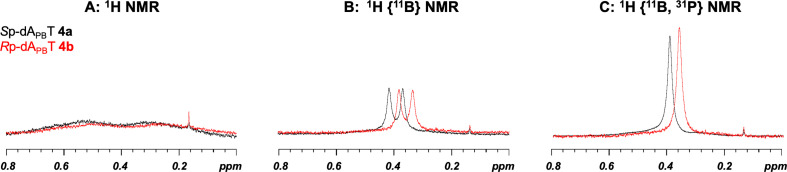
Comparison of the BH_3_ regions in the ^1^H, ^1^H {^11^B} and ^1^H {^11^B, ^31^P} NMR spectra of dA_PB_T in D_2_O at 298 K. The spectra of the *S*p and *R*p isomers are coloured black and red, respectively.

**Figure 4.**
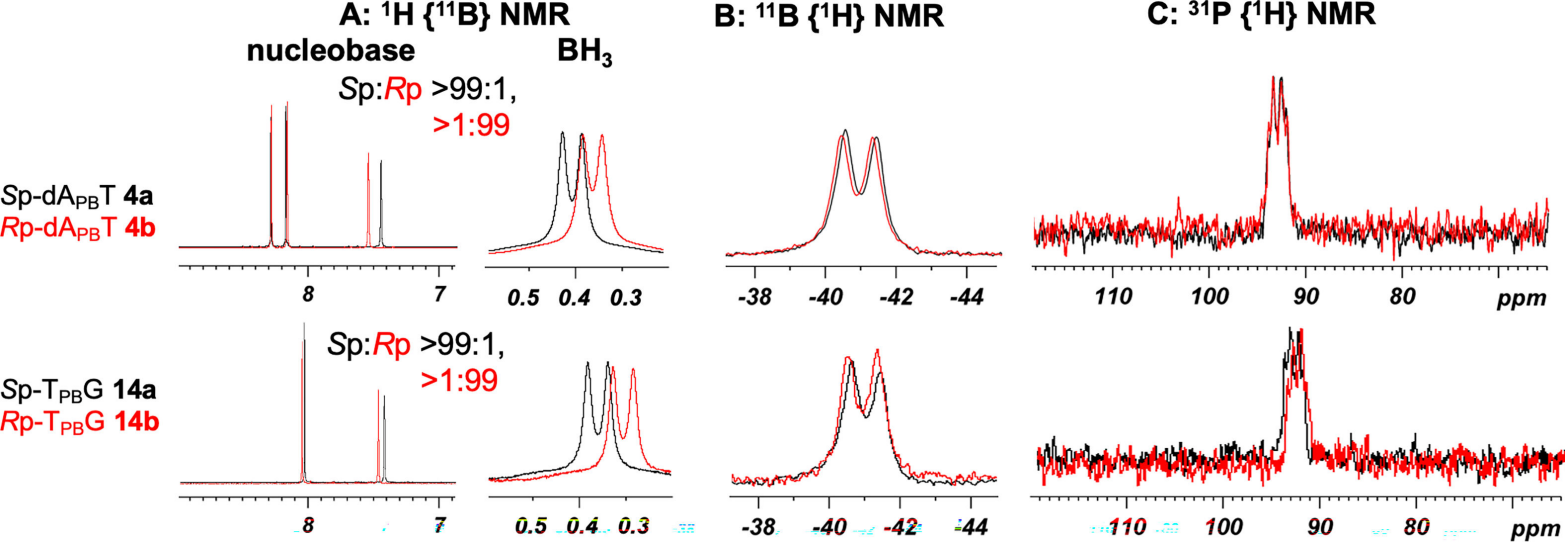
^1^H {^11^B}, ^11^B {^1^H}, and ^31^P {^1^H} NMR spectra of the dA_PB_T and T_PB_G isomers in D_2_O at 298 K. The spectra of the *S*p and *R*p isomers are coloured black and red, respectively.

**Figure 5.**
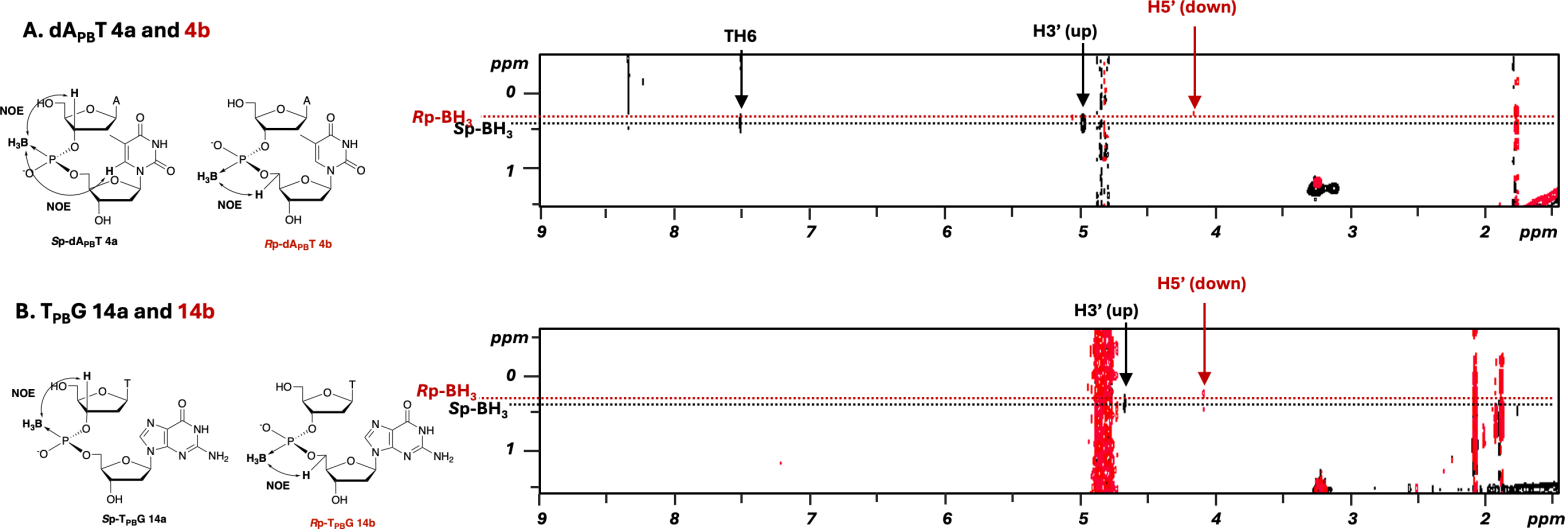
^1^H-^1^H {^11^B} NOESY (Tm = 400 ms) spectra of the dA_PB_T and T_PB_G isomers in D_2_O at 298 K. The signals of the *S*p and *R*p isomers are coloured black and red, respectively. The arrows indicate the cross peaks between the protons of the nucleosides and the protons of the BH_3_ group. For example, regarding the cross peaks of isomer **4a** indicated by the black arrow (A), a NOE correlation between the H3' atom of the 5'-upstream nucleoside (A) and the protons of the BH_3_ group was observed. In addition, a NOE correlation between the H6 atom of T and the protons of the BH_3_ group was also observed. Regarding the cross peaks of isomer **4b** indicated by the red arrow, a NOE correlation between the H5' atom of the 3'-downstream nucleoside (T) and the protons of the BH_3_ group was observed.

To elucidate the stereochemistry of the PB linkages, 10 stereopure dinucleoside PBs dA_PB_X (X = A, C, G, T) and T_PB_G were analysed by ^1^H, ^31^P and ^11^B NMR; ^1^H-^13^C heteronuclear single quantum coherence (HSQC); and ^1^H-^1^H NOESY experiments. The spectra of dA_PB_T **4a** and **4b** and T_PB_G **14a** and **14b** are shown in figures 3–5. The stereochemistry of dA_PB_T was previously determined by means of an enzyme digestion assay, and the fast-eluting and slow-eluting dimers were *S*p and *R*p isomers, respectively [12].

As shown in figure 3, the signal corresponding to the protons of the BH_3_ group in the ^1^H {^11^B, ^31^P} NMR spectra was significantly sharper than that in the ^1^H NMR spectra. To the best our knowledge, this is the first example of the analysis of PB-DNAs with both ^11^B and ^31^P decoupling by using a triple-resonance probe in ^1^H NMR experiments. Furthermore, the slight broadening of the BH_3_ signal in the ^1^H {^11^B, ^31^P} NMR spectra was attributed to coupling between the ^10^B and ^1^H atoms. The signals corresponding to the protons on the BH_3_ group of the *S*p isomer were observed at a higher field in chemical shift than those of the *R*p isomer. The high stereopurities of dA_PB_T and T_PB_G were confirmed by the signals in the nucleobase region because the chemical shifts of the protons in these nucleobases differed slightly from each other (figure 4A). In the ^31^P {^1^H} and ^11^B {^1^H} NMR spectra, the signals were broad, and the differences in the chemical shifts for each diastereomer were trivial, not allowing distinction of the diastereomers.

Next, ^1^H-^1^H NOESY experiments with ^11^B decoupling were performed for compound **4** (dA_PB_T) to observe the NOE correlations between the protons in the BH_3_ group and spatially proximate protons in the 2'-deoxyribose moiety (figure 5). The results of the NOESY experiment indicated NOE correlations between the H3' atom of the 5'-upstream nucleoside (A) and the protons of the BH_3_ group for isomer **4a** and between the H5' atom of the 3'-downstream nucleoside (T) and the protons of the BH_3_ group for isomer **4b**. In addition, a NOE correlation between the H6 atom of T and the protons of the BH_3_ group was observed for the fast-eluting isomer **4a**, whereas no NOE correlations between these groups were observed for the slow-eluting isomer **4b**.

Molecular models of the *R*p- and *S*p-dinucleoside PBs (dA_PB_T) were prepared from the corresponding dinucleoside phosphodiester, and it was determined that the BH_3_ group of the *S*p isomer was located near the H3' atom of A and the H6 atom of T (electronic supplementary material, figure S12). Conversely, the BH_3_ group of the *R*p isomer was located near the H5' atom of T. These results and the distances between the correlated atoms suggested that the fast-eluting isomer **4a** was the *S*p isomer and that the slow-eluting isomer **4b** was the *R*p isomer.

The results of the NMR experiments, molecular modelling and previously reported enzyme digestion assays [12] for the determination of stereochemistry were in good agreement, prompting us to measure the unexplored sequence of T_PB_G. Consequently, NOE correlations between the protons of the BH_3_ group and those of the sugar backbone were found to be the same as those of dA_PB_T. In the case of the fast-eluting dA_PB_A **1a**, dA_PB_C **2a** and dA_PB_G **3a**, NOE signals were observed between the protons of the BH_3_ group and the H8 atom of A, the H6 atom of C and the H8 atom of G (electronic supplementary material, figure S12). NOE signals were observed between the protons of the *S*p-BH_3_ moiety and the protons on the nucleobase of the 3'-downstream nucleoside regardless of the type of nucleobase.

To further confirm the stereochemistry, enzyme digestion assays were performed using nuclease P1 (nP1) [32] and snake venom phosphodiesterase (SVPDE) [33], which stereoselectively cleave *R*p and *S*p PB linkages, respectively [12,18,34]. Following the treatment of *S*p- and *R*p-T_PB_G with these nucleases, the products were analysed using RP-HPLC. As shown in electronic supplementary material, figure S8, isomer **14a** was resistant to nP1 but susceptible to SVPDE, whereas isomer **14b** was resistant to SVPDE but susceptible to nP1. Thus, **14a** was assigned as the *S*p isomer, and **14b** was assigned as the *R*p counterpart, consistent with the results obtained from the NOESY analysis.

**Figure 6.**
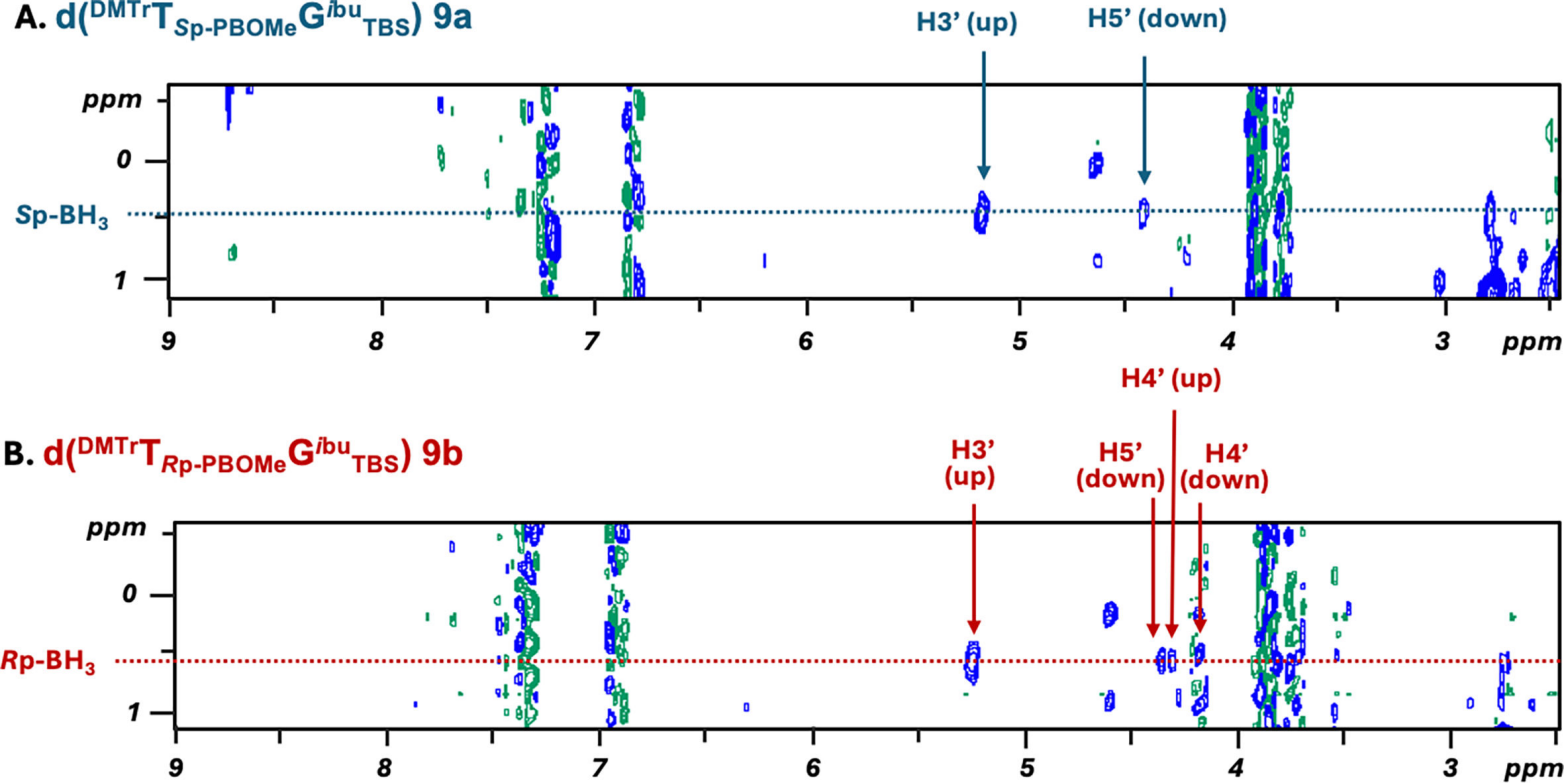
^1^H-^1^H {^11^B, ^31^P} ROESY (Tm = 200 ms) spectra of **9a** and **9b** in CDCl_3_ at 298 K. The arrows indicate the cross peaks between the protons of the nucleosides and the protons of the BH_3_ group.

To determine whether the stereochemistry of the PB linkage bearing the dimer building block was retained during the solution-phase synthesis, the stereochemistry of the fast- and slow-eluting isomers **9a** and **9b**, which were precursors of dimer building blocks **11a** and **11b**, was assigned by employing two-dimensional (2D) NMR and molecular models, as was done for dA_PB_T. In the ^1^H-^1^H ROESY experiments with ^11^B and ^31^P decoupling of the fast-eluting isomer **9a** (figure 6A), no correlation between the protons of the BH_3_ group and the H4' atom was observed. Conversely, for the slow-eluting isomer **9b** (figure 6B), correlations between the H4' atoms of both the 3'-downstream and 5'-upstream nucleosides and the protons of the BH_3_ group were observed.

A comparison of the molecular models of fully protected *R*p- and *S*p-boranophosphotriesters prepared from phosphotriesters by substituting the oxygen atom with a BH_3_ group demonstrated that the stereochemistry of **9a** and **9b** was *S*p and *R*p, respectively, indicating that the stereochemistry of the dimer building blocks was maintained during the liquid-phase synthesis of **11a** and **11b**.

#### 2.2.2. Analysis of trimers

**Figure 7.**
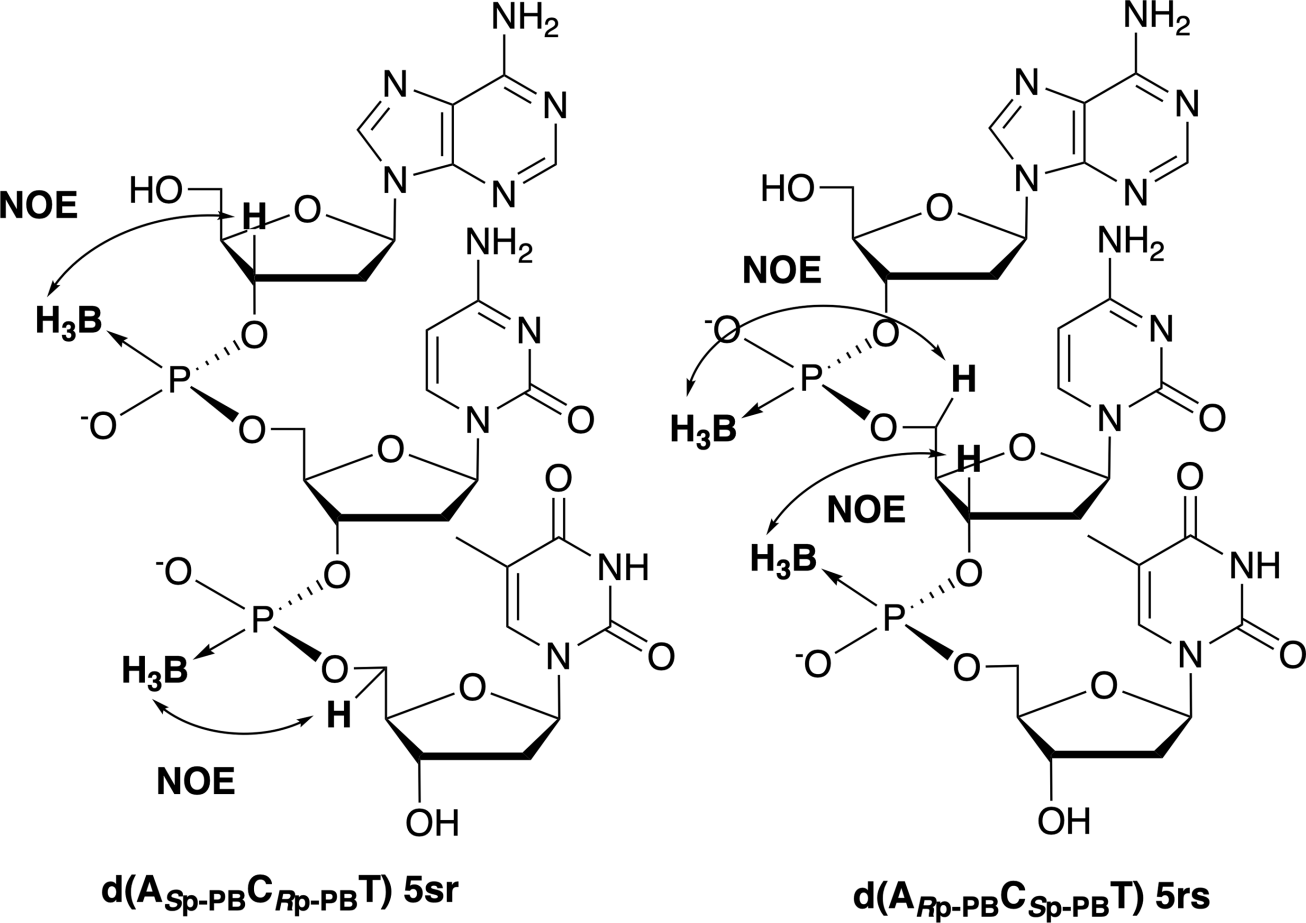
NOE correlations between the protons of the BH_3_ groups and the deoxyribose moieties of d(A*_S_*_p-PB_C*_R_*_p-PB_T) and d(A*_R_*_p-PB_C*_S_*_p-PB_T).

Next, two trimers, d(A*_S_*_p-PB_C*_R_*_p-PB_T) and d(A*_R_*_p-PB_C*_S_*_p-PB_T), containing both *S*p and *R*p PB linkages were analysed by NMR (electronic supplementary material, figures S14–S18). In the ^1^H-^1^H {^11^B} NOESY experiments for d(A*_S_*_p-PB_C*_R_*_p-PB_T), the H3ʹ atom of the 5ʹ-most upstream deoxyadenosine (A) and the H5ʹ atom of the 3ʹ-most downstream thymidine (T) were correlated with the protons of the neighbouring BH_3_ group. Conversely, in the NOESY spectrum of d(A*_R_*_p-PB_C*_S_*_p-PB_T), the H5ʹ and H3ʹ atoms of deoxycytidine (C) were correlated with the protons of the 5ʹ-upstream and 3ʹ-downstream BH_3_ groups (figure 7, electronic supplementary material, figure S17). The correlation patterns were consistent with the analysis of the dimers. These results indicated that NOESY experiments can be used to determine the stereochemistry of PB linkages in ODNs containing multiple PB linkages.

#### 2.2.3. Analysis of boranophosphate/phosphate chimeric DNA and its duplexes with complementary RNA

Finally, the stereopure PB/PO chimeric DNAs synthesized by dimer building blocks and its duplexes with complementary RNA were analysed. The assignment of the protons in the nucleobase region were conducted by NOESY experiments of the single-stranded DNAs containing a stereopure PB linkage (electronic supplementary material, figure S23), NOESY experiments of its duplexes with complementary RNA (electronic supplementary material, figures S27, S29 and S30).

**Figure 8.**
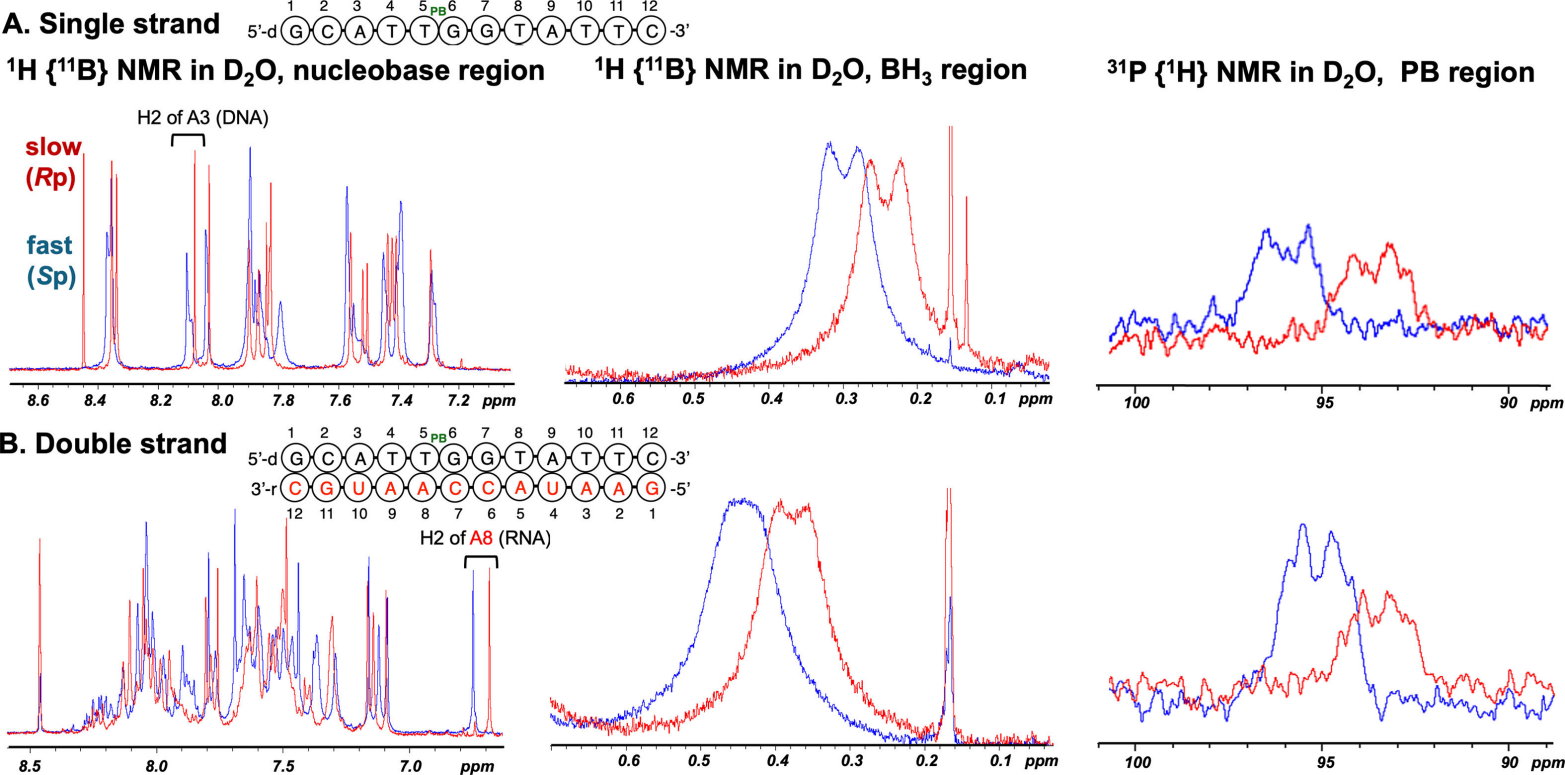
^1^H {^11^B} and ^31^P {^1^H} NMR spectra of single-stranded DNAs and DNA/RNA duplexes in D_2_O at 298 K. The spectra of the *S*p and *R*p isomers are coloured blue and red, respectively.

In the ^1^H NMR analysis of single-stranded DNAs (figure 8A), the chemical shifts of the signals of the protons on the nucleobases differed slightly between isomers. Both isomers were synthesized with a stereopurity greater than 99%, as determined from the integral ratios of the signals attributed to the H2 atom of adenine (A3H2).

**Figure 9.**
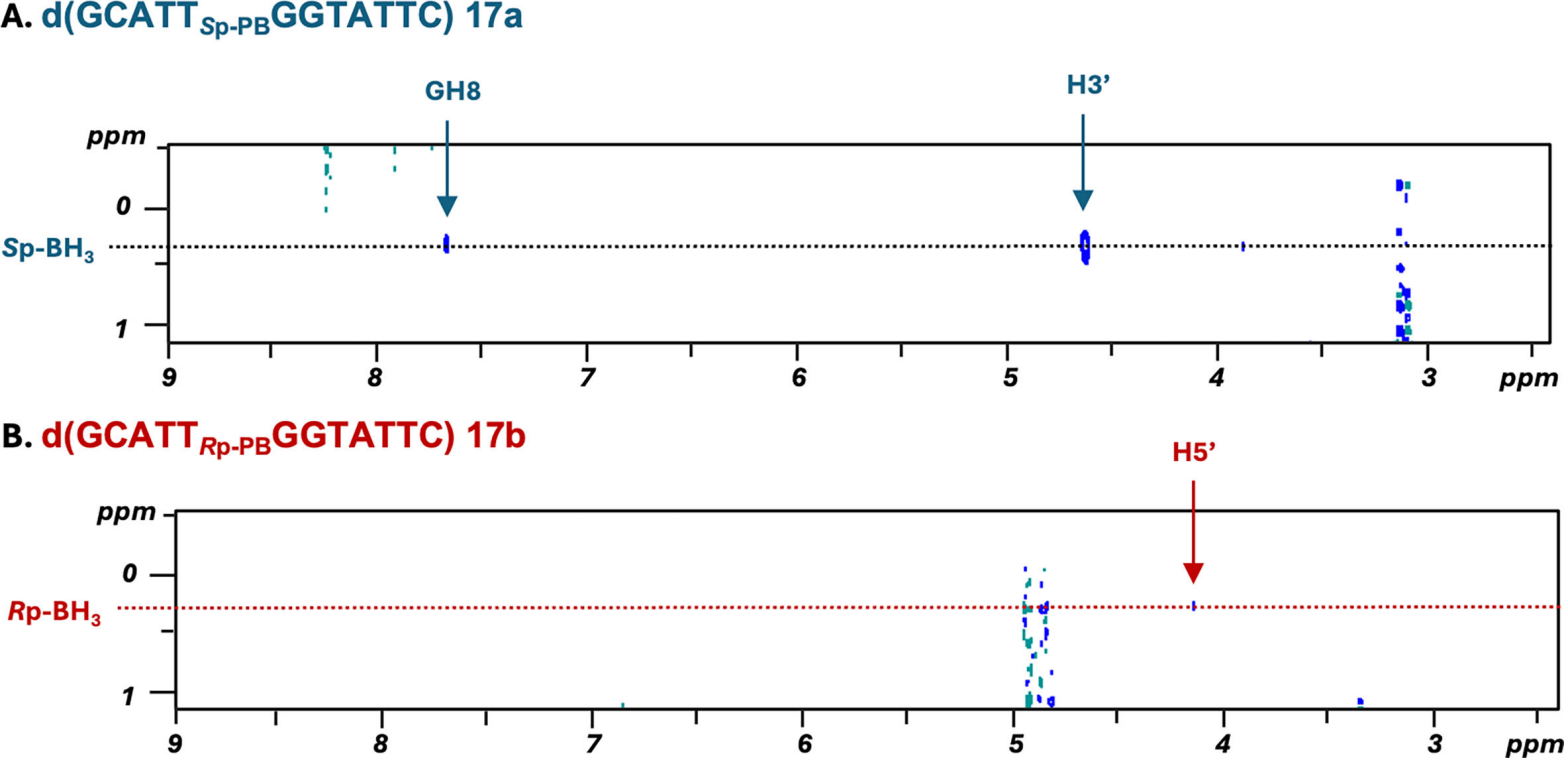
^1^H-^1^H {^11^B} NOESY spectra (Tm = 400 ms) of d(GCATT_PB_GGTATTC) isomers in D_2_O at 298 K. The spectra of the *S*p and *R*p isomers are shown in (A) and (B), respectively. The arrows indicate the cross peaks between the protons of the nucleosides and the protons of the BH_3_ group.

In the NOESY experiments, cross peaks of the protons of the BH_3_ group and the H3ʹ atom of T5 or the H8 atom of G6 were clearly observed for **17a**, whereas such cross peaks were not observed for **17b**; instead, a cross peak corresponding to the H5' atom was observed for **17b** (figure 9). The same results were observed for T_PB_G, suggesting that the stereochemistry of the T_PB_G **9a** and **9b** dimers most likely corresponded to the *S*p and *R*p configurations, respectively. These results indicated that the stereochemistry of the dimer building blocks was maintained, as determined from the analysis of T_PB_G. Therefore, this synthetic method was found to be effective for the introduction of a stereocontrolled PB linkage with the desired stereochemistry into oligonucleotides.

In the ^1^H NMR spectra of double-stranded DNA/RNA duplexes containing a single *S*p or *R*p PB linkage in the DNA strand in D_2_O, the chemical shifts of the signal of the H2 atom of adenine (A8H2) in the RNA strand differed considerably between isomers (figure 8B). It was found that the orientation of the BH_3_ group in the DNA strand affected the chemical shift of A8H2 in the RNA strand.

The following trends in the signals of the BH_3_ group in the ^1^H NMR spectra and the signals of PB in the ^31^P NMR spectra were observed: signals corresponding to the *S*p isomer tended to appear at a lower field, while those associated with the *R*p isomer were detected at a higher field. This difference in the chemical shift values between isomers may support confirmation of the stereochemistry.

NOESY experiments of double-stranded DNA/RNA duplexes in D_2_O were also conducted (electronic supplementary material, figure S26). In the NOESY spectra of the DNA/RNA duplexes containing an *S*p-PB linkage, the signals of the protons of the BH_3_ group were correlated with those of the H3ʹ atom of the nucleoside immediately upstream of the PB moiety. The correlations of the protons of the BH_3_ group and those of the deoxyribose moieties were similar to those of single-stranded DNAs. Most of the results showed the same trend as reported by Johnson *et al.* [20].

**Figure 10.**
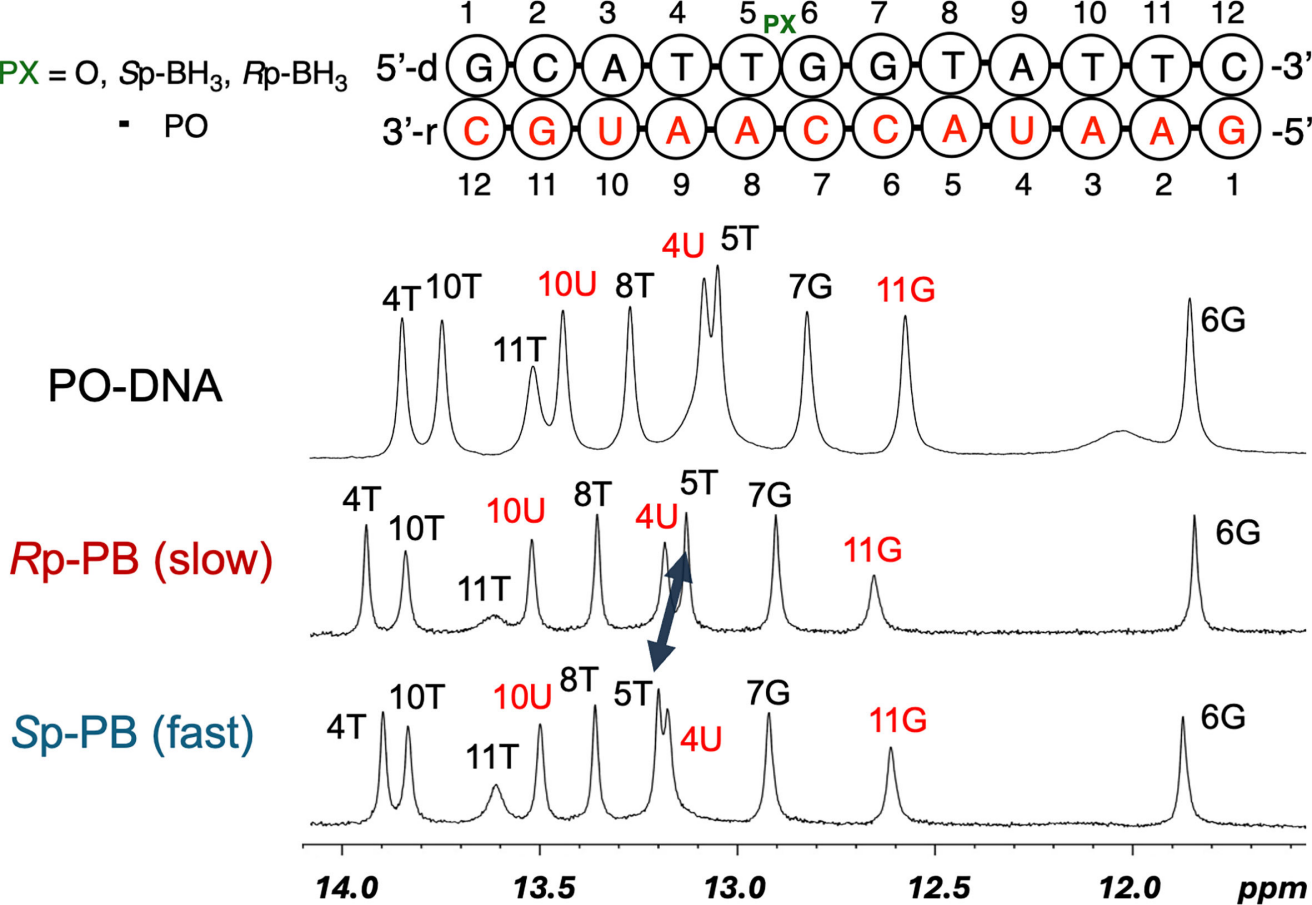
Comparison of the imino proton region of the ^1^H NMR spectra of the unmodified, *R*p-PB, and *S*p-PB DNA/RNA duplexes [10 mM NaH_2_PO_4_–Na_2_HPO_4_, 100 mM NaCl, H_2_O–D_2_O (9 : 1, v/v) pH 7.0 at 298 K]. The dark blue arrow indicates the change in the imino proton (5T) shifts between the isomers.

In the ^1^H NMR spectra of double-stranded DNA/RNA duplexes in H_2_O–D_2_O (9 : 1, v/v) (figure 10), the signals of most of the imino protons in the *R*p isomer were similar to those of the PO-DNA, but those of the T5 in the *S*p isomer differed from those of the *R*p isomer and PO-DNA. This difference was attributed to the orientation of the BH_3_ group, whether it was located inside or outside of the duplex, which may have influenced the chemical shift. The same tendency in the imino proton region of the 9-mer d(ATGGT_PB_GCTC) analysed by Johnson *et al.* was also observed [20], indicating that similar results could be obtained for samples with different sequences and lengths.

## 3. Conclusion

In this study, we synthesized PB/PO chimeric ODNs containing a stereocontrolled PB linkage using dimer building blocks with a stereodefined boranophosphotriester linkage. This strategy allowed the use of a general phosphoramidite method for the solid-phase synthesis of PB/PO chimeric ODNs, with retention of the stereochemistry and purity of the dimer building blocks. Although we synthesized only one base sequence, this strategy could be applicable for various sequences.

The stereochemistry of the phosphorus atoms in the ODNs was determined via NOESY experiments. NOE correlations between the protons of the BH_3_ group and those of the sugar moiety provided valuable information for the elucidation of the stereochemistry of the phosphorus atoms.

The synthetic and analytical method developed in this study is expected to be useful for the elucidation of the effect of stereochemistry on the physiochemical and biological properties of PB-ODNs.

## 4. Experimental section

### 4.1. Liquid-phase synthesis

All reactions were conducted under an Ar atmosphere. Organic solvents were dried according to relevant published procedures. Analytical TLC was performed on commercial glass-coated 0.25 mm thick silica gel plates.

### 4.2. Solid-phase synthesis

Solid-phase synthesis was manually conducted using a glass filter (10 × 50 mm) with a stopper at the top and a stopcock at the bottom as a reaction vessel, and the obtained compounds were analysed and purified via RP-HPLC or RP-UPLC and identified via ESI-MS (electronic supplementary material, table S1).

### 4.3. Nuclear magnetic resonance analysis

NMR spectra were measured at 298 or 283 K on a Bruker Avance Neo 500 MHz spectrometer with a double-resonance BBO cryogenic probe (Bruker BioSpin, Inc.) or a triple-resonance iTBO (Bruker BioSpin, Inc.). For the NMR processing and data analysis, Topspin 3.6.5 or 4.1.4 was used. ^1^H NMR spectra were recorded at 500 MHz using tetramethylsilane (*δ* 0.00) as an internal standard in CDCl_3_ or using MeCN (*δ* 2.06) as an internal standard in D_2_O or H_2_O–D_2_O (9 : 1, v/v). Imino proton spectra were measured in 10 mM NaH_2_PO_4_–Na_2_HPO_4_ (pH 7.0) and 100 mM NaCl in H_2_O–D_2_O (9 : 1, v/v) using the gradient pulses for water suppression, and the signals were assigned using the jump-and-return scheme [35].^13^C NMR spectra were recorded at 126 MHz in CDCl_3_, which was used as the internal standard (*δ* 77.0). ^31^P NMR spectra were recorded at 201 MHz using H_3_PO_4_ (*δ* 0.00) as the external standard in CDCl_3_ or D_2_O. Globally optimized alternating phase rectangular pulse (GARP) decoupling was used for the ^1^H {^11^B}, ^1^H {^11^B, ^31^P}, ^11^B, {^1^H} and ^31^P {^1^H} NMR experiments. Phase-sensitive gradient-enhanced (ge) 2D ^1^H-^13^C HSQC spectra were measured using echo-antiecho and adiabatic pulses for inversion and refocusing. Phase-sensitive ge−2D multiplicity-edited HSQC were measured using echo-antiecho and inversion and matched sweep adiabatic pulses. NOESY and ROESY spectra were recorded with mixing times of 400 or 200 ms, respectively.

### 4.4. Nuclear magnetic resonance samples preparation

Dimers, trimers and single-stranded DNAs were dissolved in D_2_O. Double-stranded DNA/RNAs were dissolved in D_2_O containing NMR buffer (100 mM NaCl in 10 mM NaH_2_PO_4_−Na_2_HPO_4_ buffer) or in H_2_O–D_2_O (9 : 1, v/v) containing NMR buffer. The samples were dissolved in the NMR buffer using centrifugal ultrafiltration unit vivaspin 2 (cut-off molecular weight 3000) (Sartorius AG, Germany). Then the samples were transferred in NMR tube. The amount of substance, concentration and volume of the solvent of each dissolved sample are shown in electronic supplementary material, table S2. To form DNA/RNA duplexes, the DNA and complementary RNA were simply mixed in D_2_O (200 µl) containing NMR buffer or H_2_O–D_2_O (9 : 1, v/v, 200 µl) containing NMR buffer at room temperature without annealing.

### 4.5. High-performance liquid chromatography analysis and estimation of isolated yields via UV–Vis spectra

Dimers and trimers were analysed by HPLC (LC-Net II/ADC system) using an analytical HPLC column (Delta Pak C18, 5 µm, 3.9 mm × 150 mm, Waters). RP-HPLC analysis was performed at 260 nm, 30°C and a flow rate of 0.5 m min^−1^ unless otherwise noted. Dodecamers were analysed by UPLC (ACQUITY UPLC H-Class system) using an analytical UPLC column (ACQUITY^TM^ Premier BEH C18, 1.7 µm, 2.1 mm × 50 mm, Waters). RP-UPLC measurements were performed at 260 nm, 50°C and a flow rate of 0.5 ml min^−1^ unless otherwise noted. The isolated yields of the dimers, trimers and dodecamers were estimated from the UV–Vis spectra (JASCO V−550 UV/vis spectrophotometer) using the following molar absorption constants at 260 nm: *ε* = 27 400 l mol^−1^ cm^−1^ for dA_PB_A, *ε* = 21 200 l mol^−1^ cm^−1^ for dA_PB_C, *ε* = 25 000 l mol^−1^ cm^−1^ for dA_PB_G, *ε* = 19 000 l mol^−1^ cm^−1^ for T_PB_G, *ε* = 29 000 l mol^−1^ cm^−1^ for the trimer, and *ε* = 1 13 500 l mol^−1^ cm^−1^ for the dodecamer.

### 4.6. Molecular modelling and structures calculation

Some of the structures were prepared using Discovery Studio Visualizer v. 21.1.0 (Dassault Systèmes BIOVIA Corp.) Based on Protein Data Bank (PDB) data (2lar, 2lb4) [20], the structures of the dimers and trimers were extracted and prepared. Some of the structures were evaluated by molecular mechanics calculations using MacroModel 9.1. The structure calculations were carried out using Amber* [36] as the force field parameter and water or chloroform as a solvent (GB/SA (generalized born/surface area model)) [37]. The cut-offs for non-covalent interactions in the calculations were 8 Å for van der Waals, 20 Å for Coulomb forces and 4 Å for hydrogen bonds. The calculations were carried out without setting any constraint conditions (e.g. interatomic distances, dihedral angles).

### 4.7. Enzyme digestion assay

In the SVPDE assay, SVPDE from *Crotalus adamanteus* was used. A 0.1 mM aqueous solution of **14a** or **14b** (5 μl, 0.5 nmol) and an aqueous solution of SVPDE solution (2.0 × 10^−3^ U in 45 μl) were successively added to a 200 mM Tris-HCl buffer (pH 8.0) containing 30 mM MgCl_2_ (50 μl) at 37°C. After 12 hours, the solution was heated to 95°C for 1 minute to denature SVPDE and then cooled to 4°C. The mixture was diluted with 0.1 M TEAA buffer (100 μl) and then analysed using RP-HPLC. RP-HPLC was performed with a linear gradient of 5−25% MeCN for 20 minutes in 0.1 M TEAA buffer (pH 7.0) at 30°C with a flow rate of 0.5 ml min^−1^.

In the nP1 assay, nP1 from *Penicillium citrinum* was used. A 0.1 mM aqueous solution of **14a** or **14b** (5 μl, 0.5 nmol) and an aqueous solution of nP1 solution (1 unit in 45 μl) were successively added to a 200 mM Tris-HCl buffer (pH 8.0) containing 2 mM ZnCl_2_ (50 μl) at 37°C. After 12 hours, the solution was heated to 95°C for 1 minute to denature nP1 and then cooled to 4°C. The mixture was diluted with 0.1 M TEAA buffer (100 μl) and then analysed same as condition of SVPDE assay.

## Data Availability

Our data are deposited at figshare: [37]. Supplementary material is available online [38].
